# Outcome of Atrial Fibrillation in Patients With Sepsis

**DOI:** 10.7759/cureus.19159

**Published:** 2021-10-31

**Authors:** Benish Afzal, Syed Ahsan Ali, Bushra Jamil

**Affiliations:** 1 Department of Internal Medicine, Aga Khan University Hospital, Karachi, PAK

**Keywords:** septic shock, sepsis, mortality, atrial fibrillation, arrhythmia

## Abstract

Background

Atrial fibrillation (AF) is one of the most frequent arrhythmias in critically ill patients. Sepsis is a major cause of inpatient mortality and it has been associated with cardiac arrhythmias. The objective of this study was to study the outcome of atrial fibrillation in patients who are admitted with sepsis.

Methods

This is a prospective, single-center cohort study of patients admitted to the Medicine Department between June 1, 2019, and November 30, 2019. The inclusion criteria were adult patients with sepsis and septic shock. In this study, 113 patients were enrolled and outcomes were compared between the group that developed atrial fibrillation during the hospital stay and the group without atrial fibrillation.

Results

There were 57 (50.4%) patients with AF including 23 (20.4%) who also had a prior history. Total inpatient mortality was 42 (37.2%), of which 17 patients (40.5%) had AF. AF was not found to be associated with higher mortality or increased length of hospital stay (p-value 0.103 and 0.858, respectively).

Conclusion

AF was not found to be a cause of higher mortality in patients with sepsis or septic shock. There is a need for larger-scale studies to find out the causes of high inpatient mortality in sepsis and the need for local guidelines regarding the management of AF in critically ill patients.

## Introduction

Atrial fibrillation (AF) is the most common arrhythmia worldwide, although the global burden is not known, but the estimated global age-adjusted prevalence was 0.5% in 2010, representing nearly 33.5 million individuals [1]. Many Asian epidemiological studies have shown rising trends in the prevalence of atrial fibrillation [2-5]. Actual prevalence is suspected to be higher, especially in Pakistan, as many patients are asymptomatic and remain undiagnosed [6].

Atrial fibrillation is one of the most common arrhythmias among critically ill patients [7-9]. Multiple studies have shown that the incidence of atrial fibrillation in critically ill patients varied from 1% to 44% [8,9]. There is a strong association of sepsis with cardiac arrhythmias [10]. Sepsis is characterized by a systemic release of proinﬂammatory cytokines, high levels of stress hormones, intravascular volume shifts, autonomic dysfunction, and cardiovascular compromise, all of which lead to impairment of cardiac function including its electric function, causing the development of AF [11-13].

Sepsis represents a significant proportion of morbidity and mortality in the intensive care unit. In Pakistan, a high incidence of sepsis (43.23%) was seen in patients admitted to the Surgical Intensive Care Unit (SICU) in a Karachi hospital, associated with very high mortality [14]. The development of atrial fibrillation is an indicator of poor prognosis in patients with sepsis [15]. Long-term patients with new-onset AF during sepsis may have greater risks of future occurrence of AF, heart failure, and death than do sepsis survivors without prior known AF [16,17]. There is a lack of studies showing outcomes of atrial fibrillation in patients with sepsis in the Asian population and a lack of local guidelines regarding their management. In this study, we aim to gain a better understanding of outcomes of AF in patients who present with sepsis.

## Materials and methods

This study was a prospective observational cohort study, conducted at the Internal Medicine department of Aga Khan University Hospital, a tertiary care hospital, in Karachi, Pakistan. Patients admitted to Special Care and Intensive Care Units were recruited between June 2019 to November 2019. Patients of age more than 18 years, who were admitted with sepsis or septic shock during these six months, were included in this study. Pregnant females and those patients who had implantable pacemakers were excluded. This study was approved as an exemption by the Ethics Review Committee of Aga Khan University Hospital (approval number: 2019-2007-5403). It was conducted according to the ethical guidelines of the Declaration of Helsinki. Strict confidentiality was maintained and patients’ information was anonymized and deidentified before the analysis. The informed consent of the patients was waived.

Sepsis was defined as life-threatening organ dysfunction caused by a dysregulated patient response to infection. Positive quick Sepsis-related Organ Failure Assessment (qSOFA) was used for selecting patients with sepsis. Patients who had suspected or confirmed infection on admission, with respiratory rate more than 22 breaths/min, Glasgow coma scale <15, or systolic blood pressure <100 mmHg on arrival were recruited. Septic shock was defined as patients with sepsis that require vasopressors to maintain a mean arterial pressure (MAP) of ≥ 65 mmHg and have a serum lactate level of ≥ 2 mmol/L. Demographics and clinical details of patients fulfilling inclusion criteria such as age, gender, prior history of atrial fibrillation, need for ionotropic requirements on admission, lactate levels, cultures, ECG findings, length of stay, and inpatient mortality were collected. All patients underwent continuous electrocardiographic monitoring and automatic detection of arrhythmias, as per the protocol of Special Care and Intensive Care Units. All episodes of arrhythmia were recorded and atrial fibrillation was diagnosed on 12-lead ECG.

The sample size calculation was based on the assumption that atrial fibrillation was 10% in sepsis patients [18]. By taking into account all of these figures together with a 99% confidence interval, and being exposed to non exposed ratio of 1:1, 80% power, and relative risks of 1.75, the sample size was calculated to be n=112. There were 113 patients enrolled in our database. Patients who developed atrial fibrillation were compared with patients who did not develop atrial fibrillation. Outcomes evaluated were inpatient mortality and duration of hospital stay. Statistical analysis was done with quantitative variables reported as a mean and standard deviation and categorical variables were reported as absolute numbers and percentages. Data analysis was done using SPSS Statistics for Windows, version 23.0 (IBM Corp., Armonk, NY). Data were stratified by age, gender, prior known history of atrial fibrillation, a known source of infection, duration of hospital stay, and mortality. The post-stratification Chi-square test was applied. A p-value <0.05 was considered statistically significant. To detect significant differences between groups, the Chi-square test or the Fisher’s exact test was used for categorical variables. An independent T-test was used for comparing the length of stay between the group with atrial fibrillation and the group without it.

## Results

**Figure 1:**
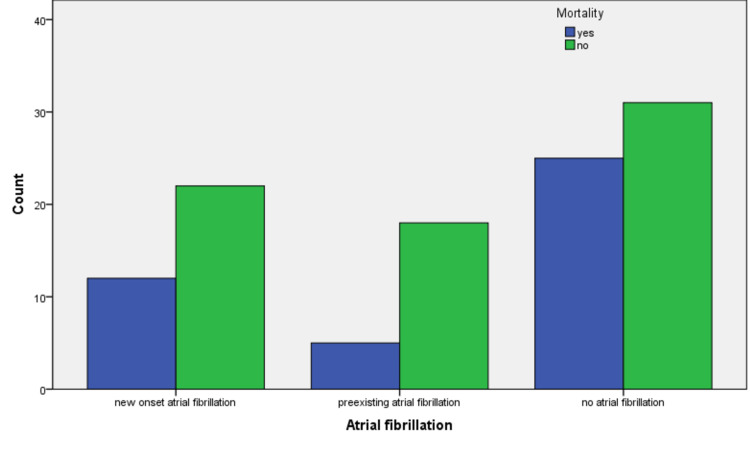
Association of atrial fibrillation (pre-existing and new-onset) with mortality

Out of 113 study participants with sepsis, 59 were male (52.2%). The mean age of the patients was 66±16.4 years. In this study, 48 (42.5%) patients developed septic shock and required vasopressor support. There were 57 (50.4%) patients who had atrial fibrillation during the hospital stay, of which 23 (20.4%) also had a history of prior atrial fibrillation. The mean length of hospital stay was 6.46±5.44 days. Inpatient mortality was 42 (37.2%), of which 17 patients (40.5%) had atrial fibrillation, as shown in Table 1 and Figure 1. According to our statistical analysis, atrial fibrillation was not found to be associated with higher mortality in patients with sepsis (p-value= 0.103). There was a 0.668 relative risk of death among these individuals with an odds ratio of 0.527.

**Table 1:** Association of atrial fibrillation with mortality in patients with sepsis CI: Confidence interval; AF: Atrial fibrillation, n: Number of patients

Patients with sepsis	Mortality		p-value	Odds ratio	95% CI
	Yes, n (%)	No, n (%)			
With atrial fibrillation during hospital stay (including new-onset AF and patients with prior history)	17 (15.0%)	40 (35.4%)	0.103	0.527	0.243-1.143
Without atrial fibrillation	25 (22.1% )	31 (27.4%)			
Total	42 (37.2%)	71 (62.8%)			

In multivariate analysis, as shown in Table 2, the hazard ratio for atrial fibrillation was 1.442 (p-value was not significant).

**Table 2:** Multivariate Cox regression (proportional hazard) analysis of factors that affect mortality in patients with sepsis

Patients' characteristics	Regression coefficient	Standard error	p-value	Hazard ratio	95% CI for the hazard ratio	
					Lower	Upper
Age	0.005	0.010	0.611	1.005	0.986	1.024
Atrial fibrillation	0.366	0.340	0.281	1.442	0.741	2.808
Vasopressor requirement during admission	-0.564	0.325	0.082	0.569	0.301	1.075

The patients were divided into two groups on basis of whether they developed atrial fibrillation or not. Length of stay was compared between these two groups, and results are shown in Tables 3, 4. The comparison in the length of stay was not found to be statistically significant (p-value 0.858).

**Table 3:** Comparison of mean length of stay of septic patients between the groups with and without atrial fibrillation n: Number of patients

		n	Mean length of stay	Standard deviation
Atrial fibrillation	Yes	57	6.37	3.745
	No	56	6.55	6.787

**Table 4:** Association of atrial fibrillation with the length of stay in patients with sepsis F: Test statistic; Sig.: Significance of test statistic; Sig. (2-tailed): p-value

Independent samples test			
	Levene's Test for Equality of Variances		T-test for Equality of Means
	F	Sig.	Sig. (2-tailed)
Length of stay	1.075	0.302	0.858

## Discussion

In this prospective, observational cohort study, we found that there is no association of atrial fibrillation with increased mortality in patients with sepsis. Larger-scale studies are needed to evaluate if this association is found in the South Asian population. Contrary to the hypothesized association there was no statistically significant difference seen in the length of stay between the two groups in this study. On the other hand, this study established that sepsis is associated with very high mortality and atrial fibrillation is a common occurrence in sepsis.

Similar results regarding the association of atrial fibrillation and inpatient mortality were found in a cohort study conducted at Firoozabad Hospital, Iran. This study included 194 intensive care unit (ICU) patients and those who developed new-onset AF (11.9%) were not found to have greater in-hospital mortality [19]. The patients with new-onset AF were found to have longer ICU stays [19].

In contrast to our study, there was a study conducted in the Netherlands to determine incidence, predictors, and outcomes of atrial fibrillation. In this study, 1,782 patients with sepsis admitted to two tertiary intensive care units were included. Atrial fibrillation occurred in 23% of individuals and it was associated with a longer stay, an increased death rate, and an overall increased mortality risk [8].

A large-scale retrospective cohort study done in the United States based on National Inpatient Sample databases (2010-2014) included 5,808,166 hospitalizations with the primary diagnosis of sepsis, of which 19.4% were associated with atrial fibrillation. In the sepsis-atrial fibrillation cohort, all-cause mortality was found to be significantly higher in sepsis patients who developed atrial fibrillation as compared to those without atrial fibrillation [20]. The greatest predictors of mortality in this group were African American race, female gender, advanced age, and the presence of medical comorbidities [20].

Many western studies have shown that atrial fibrillation is independently associated with higher mortality and morbidity [8,15,17]. This implies that in our local population there are other factors causing mortality and prolonged hospital stay in septic patients and these may include late arrival to the hospital, delayed resuscitation, antibiotic resistance, and lack of resources. A high incidence of sepsis is seen in ICU patients in Pakistan and there are poor survival rates [14].

In critically ill patients the most commonly seen arrhythmia is atrial fibrillation. In sepsis, the systemic release of proinﬂammatory cytokines causes intravascular volume shifts and autonomic dysfunction which leads to the development of AF. The negative effects of sepsis on the heart are not limited to the impairment of vascular tone and cardiac function, but they also extend to the electric function. Patients with sepsis may be more prone to hemodynamic decompensation during episodes of AF. Management of AF in septic patients depends on multiple factors including the hemodynamic status of the patient, electrolyte imbalances, and presence of any offending agents. In these patients, the presence of AF complicates management as patients require vasopressor support which may worsen arrhythmias. These patients also require rate control or rhythm control and decisions regarding anticoagulation.

The limitations of our study were the small sample size and inclusion of both ICU and non-ICU patients. In our study, we included all patients presenting with sepsis or septic shock regardless of their source of infection, which could be a confounding factor. It is beyond the scope of this study to access post-discharge mortality, readmissions, or persistence of atrial fibrillation after discharge.

There is a need for randomized trials to see if the management of atrial fibrillation should be different in septic patients and to find which medications should be preferred to improve prognosis. There is a high burden of sepsis which requires improvement in our resuscitation attempts, local antibiotics guidelines, and working on measures for source control. Local surveys should be conducted to find out the causes for delays in hospital arrival.

## Conclusions

In this study association of atrial fibrillation with mortality or longer hospital stay in sepsis patients was not proven; however, there is a need for larger-scale studies. There is an urgent need to evaluate the causes of high mortality in patients with sepsis in our population and to study possible ways to reduce it. AF may be considered as a presentation of organ dysfunction caused by sepsis. It is the most common arrhythmia in critical care settings and patients who develop it should be closely monitored. There is a need for clinical trials and focused local guidelines for the management of AF in patients with sepsis and septic shock.
